# Action of the Bromide of Potassium

**Published:** 1867-06

**Authors:** 


### Action of the Bromide of Potassium.

The actions of the bromide of potassium, according to Dr. J. Crichton Browne, in the *American Journal of the Medical Sciences*, are—(1.) It mitigates those convulsive movements of spasmodic twitchings, which are the result of the rapid conversion of censory impressions in motor impulses, or of morbid reflex action through the medulla oblongata, and it exercises a peculiar influence over the phenomena which are characteristic of epilepsy. Whether the increased excitability of the medulla oblongata is so great as to be productive of epilepsy, or so slight as so expend itself in minor spasmodic complaints, the bromide seems to exert an excellent effect on it. (2.) It has a sedative effect upon the action of the heart in certain cases. (3.) It lessens and mitigates the rapid and preternatural excitement of the spasm, tremor, and other outward manifestations, which in some forms of nervous disease follow upon any emotional or moral disturbance. (4.) It acts as an anodyne, under certain circumstances, relieving hyperæsthetical sensations. (5.) It produces sleep. (6.) It exercises a beneficial influence over certain mental diseases.—*The Druggists' Cir. and Chem. Gazette.*

